# Supplementary material for: Social and natural risk factor correlation in China's fresh agricultural product supply
Source: PLoS One. 2020 Jun 2;15(6):e0232836. doi: 10.1371/journal.pone.0232836 (PMC7266298; doi:10.1371/journal.pone.0232836)
Supplement: Attachment — Submitted filename: Response to Reviewers.docx [file pone.0232836.s001.docx]

Response to Reviewers

Dear Editor:

Thank you for your letter and the comments concerning our manuscript entitled “Social and Natural Risk Factors Correlation in China's Fresh Agricultural Products Supply”. Those comments are all valuable and very helpful for revising and improving our paper, as well as the important guiding significance to our researches. We have studied comments carefully and have made correction which we hope meet with approval. The main corrections in the paper and the responds are as flowing:

In the Editors’ Comments

Some of the major weaknesses of the paper that stand out:

1) the cover letter should be rewrote. First of all, the journal you submitted is PLOS ONE, not "“System Dynamics Review”. And, please spell the journal name in a right way, it should be "PLOS" instead of "POLS". Hope you never make this mistake again.

Response: This is a serious mistake, and we have rewritten the letter, hoping to get your forgiveness.

2) the order of the second Affiliation should be "School of Business, Yangzhou University" instead of "Yangzhou University School of Business", please check it carefully.

Response: We created a Title Page document and wrote it according to the template(<https://journals.plos.org/plosone/s/file?id=7797/Title%20Page%20-%20ONE%20Formatting.pdf>) provided by PLOS ONE. The following is a partial interception of the modified content:

^1^School of Management, Jiangsu University, Zhenjiang, Jiangsu, China

^2^School of Business, Yangzhou University, Yangzhou, Jiangsu, China

Other changes can be reviewed in the Title Page document.

3) a clear statement and describition of the selection process regarding the risk factors is needed in section 4.2

Response: We rewrite the selection process for risk factors and make the statement clearer by listing the selection steps.

(1) Collecting relevant data for textual analysis, extracting information according to keyword risk and failure, and determining the initial risk factors.

(2) Identifying members of the expert group.

(3) Gathering individual opinions on the initial risk factors and analyzing these opinions from the expert group.

(4) Compiling information on new comprehensive opinions and sending to each members of the expert group for review.

(5) Asking each expert to revise their own opinions based on the responses from the expert group. If the individual's response is significantly different from that of the group norm, the member needs to provide a rationale for his differing viewpoint.

(6) Analyzing the new individual opinions and returning to the members the responses.

(7) This analysis process is repeated many times to gradually obtain a more consistent result.

4) please read the "Guidelines for authors" carefully and make sure all the formats meet the requirement of PLOS ONE. for example, there should be "1, 2, 3……“ after "5. Research methods", you can use either "(1), (2)" or "5.1"

Response: we read the "Guidelines for authors" (<https://journals.plos.org/plosone /s/file?id=4497/Main%20Body%20-%20ONE%20Formatting.pdf>) carefully and make sure all the formats meet the requirement of PLOS ONE. For example, we re-edited the format of headings, references, and table headings.

In the Journal Requirements

1. Please ensure that your manuscript meets PLOS ONE's style requirements, including those for file naming. The PLOS ONE style templates can be found at <http://www.plosone.org/attachments/PLOSOne_formatting_sample_main_body.pdf> and <http://www.plosone.org/attachments/PLOSOne_formatting_sample_title_authors_affiliations.pdf>.

Response: The two URLs given here cannot be opened. We found the main body and title page format requirements in the PLOS ONE’s submission guidelines, and we read the guidelines (<https://journals.plos.org/plosone/s/file?id=4497/Main%20Body%20-%20ONE%20Formatting.pdf> and <https://journals.plos.org/plosone/s/file?id=7797/Title%20Page%20-%20ONE%20Formatting.pdf>) carefully and make sure all the formats meet the requirement of PLOS ONE.

2. We suggest you thoroughly copyedit your manuscript for language usage, spelling, and grammar. If you do not know anyone who can help you do this, you may wish to consider employing a professional scientific editing service.

Response: We took your suggestion and used AJE's language polishing service.

The service certificate is as follows:

Language changes can be viewed in the new manuscript.

3. We noticed you have some minor occurrence of overlapping text with the following previous publication(s), which needs to be addressed (<https://doi.org/10.1016/j.frl.2020.101491>). Your revision ensure you cite all your sources (including your own works), and quote or rephrase any duplicated text outside the methods section. Further consideration is dependent on these concerns being addressed.

Response: we added the previously missed reference and details as follows:

23. Yu, S., Lizhen, C., Huaping, S., Farhad, T. H.. Low-carbon financial risk factor correlation in the belt and road PPP project. [Finance Research Letters](https://www.sciencedirect.com/science/journal/15446123). 2020 [3 July 2020], Available from: https://doi.org/10.1016/j.frl.2020.101491.

We tried our best to improve the paper and made changes in the manuscript. We appreciate for your warm work earnestly, and hope that the correction will meet with approval.

Sincerely yours, Xiaoyu Bian
