# Supplementary material for: Social and natural risk factor correlation in China's fresh agricultural product supply
Source: PLoS One. 2020 Jun 2;15(6):e0232836. doi: 10.1371/journal.pone.0232836 (PMC7266298; doi:10.1371/journal.pone.0232836)
Supplement: Attachment — Submitted filename: Review report PLOS ONE.docx [file pone.0232836.s002.docx]

Dear Editor,

The manuscript entitled “Social and Natural Risk Factor Correlation in China's Fresh agricultural product supply” has some strengths. This topic is fascinating, considering the increasing importance of social and natural risk factors in the agricultural sector. The paper is well written and easy to follow.

The authors have made a significant effort in revising the paper. They have solved almost all the comments. The paper can be accepted for publication in its current form.

**Sincerely**

**Dhekra Ben Amara**
