# Supplementary material for: Social and natural risk factor correlation in China's fresh agricultural product supply
Source: PLoS One. 2020 Jun 2;15(6):e0232836. doi: 10.1371/journal.pone.0232836 (PMC7266298; doi:10.1371/journal.pone.0232836)
Supplement: Attachment — Submitted filename: Response to Reviewers.docx [file pone.0232836.s004.docx]

Response to Reviewers

Dear Editor and Reviewers:

Thank you for your letter and the comments concerning our manuscript entitled “Social and Natural Risk Factors Correlation in China's Fresh Agricultural Products Supply”. Those comments are all valuable and very helpful for revising and improving our paper, as well as the important guiding significance to our researches. We have studied comments carefully and have made correction which we hope meet with approval. The main corrections in the paper and the responds are as flowing:

In the Reviewers’ Comments

1) Reviewer #1: The manuscript entitled “Social and Natural Risk Factor Correlation in China's Fresh agricultural product supply” has some strengths. This topic is fascinating, considering the increasing importance of social and natural risk factors in the agricultural sector. The paper is well written and easy to follow.The authors have made a significant effort in revising the paper. They have solved almost all the comments. The paper can be accepted for publication in its current form.

Response: Thank you for your recognition of this manuscript entitled “Social and Natural Risk Factor Correlation in China's Fresh agricultural product supply”, we are very pleased that the article has been approved by experts.

2) Reviewer #2: Modification comments displayed on the [view attachments](https://www.editorialmanager.com/pone/author_ViewRevAttach.asp%3Fdocid%3D538448%26ms_num%3DPONE-D-20-07597%26rev%3D1)  entitled “PLOS Manuscript”.

Response: Thank you for your careful review of the manuscript entitled “Social and Natural Risk Factor Correlation in China's Fresh agricultural product supply”. We have revised all the language issues and format errors in the manuscript you raised, and see the modified version for details.

We tried our best to improve the paper and made changes in the manuscript. We appreciate for your warm work earnestly, and hope that the correction will meet with approval.

Sincerely yours, Xiaoyu Bian
